# Co-Occurrence of Rare ArmA-, RmtB-, and KPC-2–Encoding Multidrug-Resistant Plasmids and Hypervirulence *iuc* Operon in ST11-KL47 Klebsiella pneumoniae

**DOI:** 10.1128/spectrum.02371-21

**Published:** 2022-03-24

**Authors:** Ying Zhou, Wenxiu Ai, Yinjuan Guo, Xiaocui Wu, Bingjie Wang, YanLei Xu, Lulin Rao, Huilin Zhao, Xinyi Wang, Fangyou Yu

**Affiliations:** a Department of Clinical Laboratory Medicine, Shanghai Pulmonary Hospital, Tongji University School of Medicine, Shanghai, People’s Republic of China; b Department of Respiratory Medicine, First Affiliated Hospital of Wenzhou Medical University, Wenzhou, People’s Republic of China; Houston Methodist Hospital

**Keywords:** *K. pneumoniae*, plasmid, *rmtB*, *bla*_KCP-2_, *armA*, *iuc* operon

## Abstract

The rapid emergence of carbapenem-resistant Klebsiella pneumoniae (CRKP) and the comparatively limited development of new antibiotics pose a major threat to public health. Aminoglycosides are important options that can lower the mortality rate effectively in combination therapy with β-lactam agents. However, in this study, we observed two multidrug-resistant (MDR) K. pneumoniae named 1632 and 1864 that exhibited high-level resistance to both carbapenems and aminoglycosides. Through whole-genome sequencing (WGS), the unusual co-occurrence of *rmtB*, *armA*, and *bla*_KPC-2_ genes, associating with two key resistance plasmids, was observed in two isolates. Notably, we also found that the *armA* resistance gene and virulence factor *iuc* operon co-occurred on the same plasmid in K. pneumoniae 1864. Detailed comparative genetic analysis showed that all these plasmids were recognized as mobilizable plasmids, as they all carry the essential *oriT* site. Results of conjugation assay indicated that *armA*-positive plasmids in two isolates could self-transfer to Escherichia coli J53 effectively, especially, the p1864-1 plasmid, which could cotransfer hypervirulent and multidrug-resistant phenotypes to other isolates. Moreover, multiple insertion sequences (ISs) and transposons (Tns) were also found surrounding the vital resistant genes, which could even form a large antibiotic resistance island (ARI) and could stimulate mobilization of resistant determinants. Overall, we report the uncommon coexistence of *armA* plasmid, *rmtB-bla*_KPC-2_ plasmid, and even *iuc* virulence operon-encoding plasmid in K. pneumoniae isolates, which greatly increased the spread of these high-risk phenotypes and which are of great concern.

**IMPORTANCE** Carbapenemase-producing Klebsiella pneumoniae have become a great challenge for antimicrobial chemotherapy, while aminoglycosides can lower the mortality rate effectively in combination therapy with them. Unfortunately, we isolated two K. pneumoniae from blood sample of patients that not only exhibited high-level resistance to carbapenems and aminoglycosides but also showed the unusual co-occurrence of the *rmtB*, *armA*, and *bla*_KPC-2_ genes. These elements were all located on mobile plasmids and flanked by polymorphic mobile genetic elements (MGEs). What’s worse most, we also identified a conjugative virulent MDR plasmid, coharboring multiple resistant determinants, and *iuc* operon, which was confirmed could transfer such high-risk phenotype to other isolates. The emergence of such conjugative virulence plasmids may promote the rapid dissemination of virulence-encoding elements among Gram-negative pathogens. This uncommon coexistence of *rmtB*, *armA*, *bla*_KPC-2_, and *iuc* virulence operon-encoding plasmids in K. pneumoniae, presents a huge threat to clinical treatment. Future studies are necessary to evaluate the prevalence of such isolates.

## INTRODUCTION

Carbapenemase-producing Klebsiella pneumoniae (CRKP) have emerged as a great threat to public health because of the extremely limited antibiotic therapy options (1, 2). Aminoglycosides are important options for treating life-threatening infections caused by CRKP, and the combination with β-lactam agents could effectively lower the mortality rate (3, 4). However, the high-level aminoglycoside resistance phenotype has been observed in K. pneumoniae recently, which greatly enhanced the therapy trouble, leading to a global concern (5).

Most aminoglycoside resistance mechanisms were associated with the aminoglycoside-modifying enzymes (AMEs) (6), including acetyltransferases (AACs), nucleotidyltransferases (ANTs), and phosphotransferases (APHs), among which the ability to modify aminoglycosides was different. In addition, overexpression of the specific efflux pumps also contributes to aminoglycoside resistance (7), and 16S rRNA methyltransferase (16S RMTase)-encoding genes could mediate high-level resistance to aminoglycosides (8). Since the 16S RMTases encoding genes were first discovered in *Enterobacteriaceae* and Pseudomonas aeruginosa in 2003, these genes have been identified globally in multiple Gram-negative organisms. Until now, we have found ten 16S RMTase-encoding genes (*armA*, *rmtA* to *rmtH*, *npmA*, and *npmB*), which confer high-level resistance to all clinically relevant aminoglycosides (minimal inhibitory concentration [MIC], >256 mg/L) (8, 9). Among these genes, *rmtB* and *armA* present the most widespread 16S rRNA methylase genes (10, 11). Notably, genes encoding methylases can be carried in integrons or transposons located in a variety of plasmids and sometimes along with those for extended spectrum β-lactamases (ESBLs), carbapenemases, and fluoroquinolone resistance determinants, which may not only facilitate the rapid spread of 16S-RMTase genes but also could render ineffective multiple classes of antimicrobials used to treat multidrug-resistant Gram-negative infections (12).

Although the 16S RMTase-encoding genes, especially *rmtB* and *armA*, were widely present in *Enterobacteriaceae*, the coexistence of two or more such genes was unusual. Previous studies found that the two copies of *armA* combined with six AME genes in one isolate could mediate an extremely high aminoglycosides resistance level (>1,024 mg/L) (13). These results indicated that the co-occurrence of multiple aminoglycosides resistance genes may result in a higher resistance phenotype.

In this study, we found two multidrug-resistant K. pneumoniae isolated from two clinical patients that exhibited high-level resistance to both aminoglycosides and carbapenemases. We applied whole-genome sequencing (WGS) to discover the potential molecular mechanisms and did a detailed analysis of the related resistance and virulence determinants to further characterize the threat of such K. pneumoniae; then we applied the corresponding experiments to confirm the relevant phenotype. Moreover, we also analyzed the plasmid-backbone and conjugation modules to determine the potential movability of these resistant genes and applied a conjugation assay to further determine the self-transmissibility of these resistance genes. In addition to the plasmids, we described other mobile genetic elements (MGEs) flanked with the resistant genes, through the genetic comparisons as well. Overall, our goal was to report and describe clinical multidrug-resistant K. pneumoniae clearly and emphasize the possible risk of these strains.

## RESULTS

### K. pneumoniae 1632 and 1864 exhibited high-level resistance to carbapenems and aminoglycoside.

In order to clarify the antibiotic-resistant phenotypes of K. pneumoniae 1632 and 1864, we tested the susceptibility of 24 antibiotics in these strains (Table 1). We found that K. pneumoniae 1632 and 1864 posed similar multidrug-resistant features; they exhibited high-level resistance to all β-lactam antibiotics, including carbapenems, and aminoglycoside antibiotics but were still susceptible to ceftazidime-avibactam and tigecycline. These antibiotic-resistant phenotypes were not common in carbapenem-resistant *Enterobacteriaceae* (CRE); hence, we applied WGS to further explore the molecular resistance mechanisms of such two MDR isolates.

**TABLE 1:** Antimicrobial drug susceptibility profiles^*a*^

Antibiotics	MIC (mg/L)/antimicrobial susceptibility				
	K. pneumoniae 1632	K. pneumoniae 1864	E. coli J53	Transconjugants	
				p1632-2-J53 (*armA*)	p1864-1-J53 (*armA* and *iuc* operon)
MEM	>16/R	>16/R	≤0.06/S	≤0.06/S	≤0.06/S
IPM	16/R	16/R	≤0.25/S	≤0.25/S	≤0.25/S
ETP	>2/R	>2/R	≤0.015/S	≤0.015/S	≤0.015/S
GEN	>1024/R	>1024/R	≤1/S	1024/R	1024/R
AMK	>1024/R	>1024/R	≤16/S	1024/R	1024/R
AMP	>32/R	>32/R	≤8/S	>32/R	>32/R
CZO	>32/R	>32/R	≤2/S	>32/R	>32/R
CAZ	>128/R	>128/R	≤0.25/S	1/S	1/S
FEP	>16/R	>16/R	≤0.5/S	4/SDD	8/SDD
CSL	>64/32/R	>64/32/R	≤16/8/S	≤16/8/S	≤16/8/S
SAM	>32/16/R	>32/16/R	≤16/4/S	≤16/4/S	16/8/I
FOX	>32/R	>32/R	≤8/S	≤8/S	≤8/S
CXM	>16/R	>16/R	8/S	>16/R	>16/R
CTX	>64/R	>64/R	≤0.12/S	32/R	32/R
TZP	>128/4/R	>128/4/R	≤16/4/S	≤16/4/S	≤16/4/S
AMC	>32/16/R	>32/16/R	≤8/4/S	≤8/4/S	≤8/4/S
LVX	>8/R	>8/R	≤0.12/S	≤0.12/S	≤0.12/S
MFX	>2/R	>2/R	≤0.25/S	≤0.25/S	≤0.25/S
TCY	≤2/S	≤2/S	≤2/S	≤2/S	≤2/S
ATM	>16/R	>16/R	≤4/S	≤4/S	≤4/S
NIT	>64/R	>64/R	≤16/S	≤16/S	≤16/S
SXT	>4/76/R	≤0.5/9.5/S	≤0.5/9.5/S	>4/76/R	>4/76/R
Caz/AVI	2/4/S	2/4/S	≤0.5/4/S	≤0.5/4/S	≤0.5/4/S
TGC	0.5/S	0.5/S	≤0.25/S	≤0.25/S	≤0.25/S

*^a^*MIC, minimal inhibitory concentration; MEM, meropenem; IPM, imipenem; ETP, ertapenem; Caz/AVI, ceftazidime-avibactam; TGC, tigecycline; FOS, fosfomycin; AMP, ampicillin; CZO, cefazolin; CAZ, ceftazidime; FEP, cefepime; CSL, cefoperazone/sulbactam; SAM, ampicillin/sulbactam; FOX, cefoxitin; CXM, cefuroxime; CTX, cefotaxime; TZP, piperacillin-tazobactam; AMC, amoxicillin/clavulanic acid; LVX, levofloxacin; MFX, moxifloxacin; TCY, tetracycline; GEN, gentamicin; AMK, amikacin; ATM, aztreonam; NIT, nitrofurantoin; SXT, trimethoprim/sulfamethoxazole.

### K. pneumoniae 1632 and 1864 coharboring *armA*, *rmtB*, and *bla*_KPC-2_.

According to the WGS analysis, we found that both K. pneumoniae 1632 and 1864 belonged to ST11-KL47 isolates, which the pandemic of CRKP is mainly associated with. We found more than ten resistant elements and two resistance plasmids in these isolates (Table 2). Moreover, three key resistance genes were focused on that played a significant role in the formation of resistance to carbapenems (*bla*_KPC-2_), and aminoglycoside (*armA* and *rmtB*). Notably, although both *armA* and *rmtB* were general determinants that mediated the aminoglycoside resistance, the coexistence of both of them in one isolate was unusual. Moreover, *bla*_KPC-2_ and *rmtB* were found colocated on one IncFII plasmid, which indicated that there was possibility of cotransmission of these two resistant elements.

**TABLE 2:** General features and antimicrobial resistance genes of plasmids in K. pneumoniae 1632 and 1864

Characteristics	1632				1864			
	p1632-1	p1632-2	p1632-3	p1632-4	p1864-1	p1864-2	p1864-3	p1864-4
Accension no.	CP084498	CP084499	CP084500	CP084501	CP084493	CP084494	CP084495	CP084496
Length (bp)	163993	91703	10060	5596	296908	152725	43518	11970
GC content (%)	54	51	55	51	48	55	35	56
No. of ORF^*a*^	209	124	17	12	324	197	63	17
Incapability group	IncFII/IncR	IncM2	ColRNAI	/^*b*^	IncFIB/IncHIB	IncFII/IncR	/	ColRNAI
Conjugal ability								
* OriT* (start…stop) (bp)	34258.0.34343	68441.0.68546	/	/	125695.0.125722	40426.0.40511	/	/
* *Relaxase (start…stop) (bp)	153996.0.158669	68852.0.70831	/	/	46046.0.48994	/	/	/
* *T4CP (start…stop) (bp)	151780.0.153996	29046.0.31133	/	/	43930.0.46056	/	/	/
* *T4SS (start…stop) (bp)	33691.0.47918	70845.0.90445	/	/	43930.0.66313	39859.0.52106	/	/
	63667.0.67498				212221.0.246796			
	140857.0.160310							
Resistant genes								
	*rmtB*	*armA*	/	/	*armA*	*rmtB*	/	/
	*bla*_KPC-2_	*bla*_TEM-1B_	/	/	*bla*_TEM-1B_	*bla*_KPC-2_	/	/
	*bla*_SHV_	*aac(3)-IId*	/	/	*aac(3)-IId*	*bla*_SHV_	/	/
	*bla*_TEM-1B_	*bla*_CTX-M-15_	/	/	*dfrA12*	*bla*_CTX-M-65_	/	/
		*dfrA12*	/	/	*aadA2*	*bla*_TEM-1B_	/	/
		*aadA2*	/	/	*sul1*	*fosA3*	/	/
		*sul1*	/	/	*msr(E)*	*bla*_CTX-M-44_	/	/
		*msr(E)*	/	/	*mph(E)*		/	/
		*mph(E)*	/	/	*qnrB4*		/	/
					*bla*_DHA_			
Virulence factors	/	/	/	/	*iucABCD/iutA*	/	/	/

*^a^*ORF, open reading frame.

*^b^*/, no such information.

In addition to the resistance elements, we also observed that a key virulent factor, the *iutA-iucABCD* operon, was coharbored with *armA*, *bla*_TEM-1B_, *aac(3)-IId*, *dfrA12*, *aadA2*, *sul1*, *msr(E)*, *mph(E)*, q*nrB4*, and *bla*_DHA_, on the p1864-1 plasmid (K. pneumoniae 1864). We also detected the siderophore production of K. pneumoniae 1864 and 1632 to determine the virulence contribution of the *iutA-iucABCD* operon, which was highly related to K. pneumoniae virulence phenotype (14), in K. pneumoniae 1864. We found a significant increase of siderophore production in K. pneumoniae 1864, compared with 1632, which did not harbor the *iuc* operon.

### *armA* gene could transfer at high level from K. pneumoniae 1632 to recipients.

**FIG 1:**
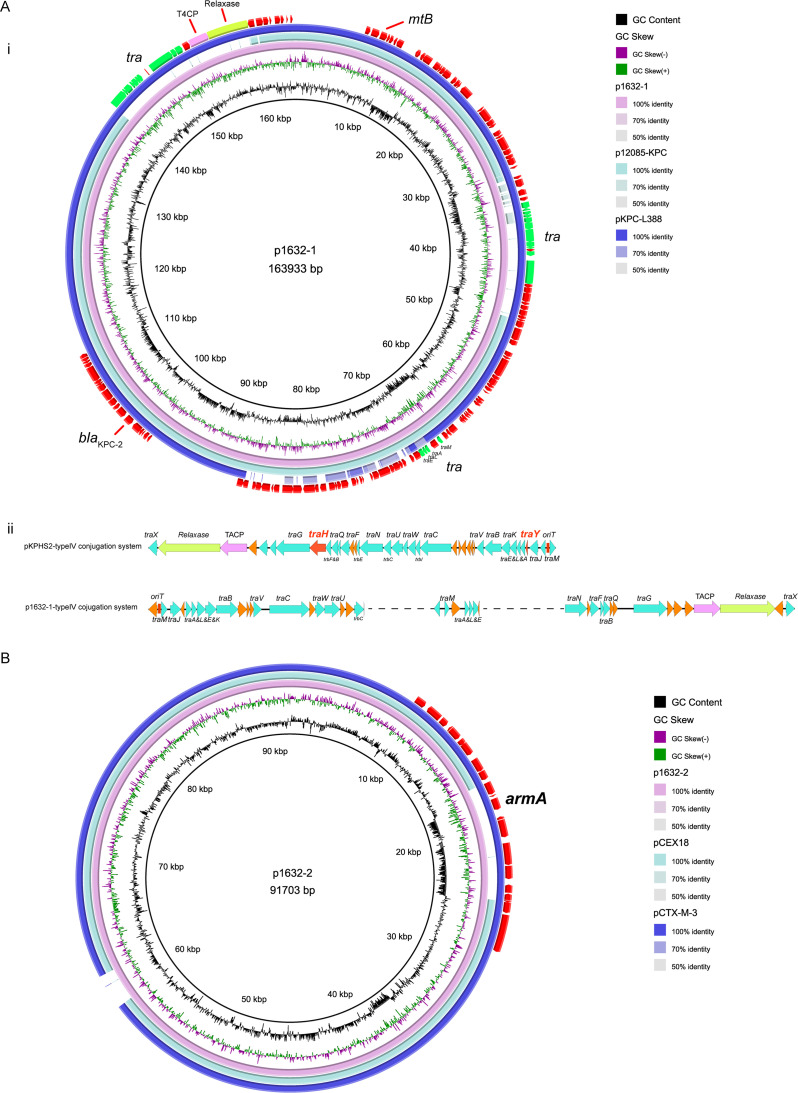
Comparative analysis of p1632-1 and p1632-2 plasmids with other reference plasmids. (A) (i) p1632-1 (CP084498) was used as the reference plasmid to perform genome alignment with two IncFII plasmid (harboring *rmtB* and *bla*_KPC_): p1285-KPC (MN842292.1) and pKPC-L388 (CP029225.1). (ii) Comparison of the conjugative system of the reference plasmid pKPHS2 (CP003224.1) and p1632-1. (B) p1632-2 (CP084499) was used as the reference plasmid to perform genome alignment with other IncM2 plasmid pEX18 (LC556221.1) and pCTX-M-3 (AF550415.2). tra, the cluster region of tra protein.

In 1632, we observed two resistant plasmids: p1632-1 (163,993 bp) and p1632-2 (91,703 bp) (Fig. 1). p1632-1 was an IncFII-type plasmid and shared high identity with two IncFII plasmids (harboring *rmtB* and *bla*_KPC-2_), p1285-KPC (MN842292.1) and pKPC-L388 (CP029225.1) (Fig. 1A). The key genetic difference of these three IncFII plasmids was the conjugation system module. Notably, we did not observe whether the p1632-1 plasmid could transfer to the J53 recipients, although it also had conjugative modules. We selected the classical high-conjugative IncFII *bla*_KPC-2_ plasmid pKPHS2 (CP003224.1) as the reference to analyze the conjugative systems of p1632-1 plasmid (*bla*_KPC-2_) to discover the potential mechanisms. We found that compared to the conjugative system of pKPHS2, the system in p1632-1 could be divided into three discontinuous parts. Moreover, such a system in p1632-1 also lacked two key type 4 secretion system (T4SS)-associated proteins, TraH, and TraY, which make a significant contribution to F pilus assembly (Fig. 1B). The deletion of key functional proteins may explain the lack of self-transmission of the p1632-1 plasmid.

p1632-2 belonged to IncM2 type plasmid and was also highly identical (99.9%) to another two IncM2 plasmids: pEX18 (LC556221.1, K. pneumoniae) and pCTX-M-3 (AF550415.2, Citrobacter freundii) (Fig. 1B). The main difference between p1632-2 and pEX18 is the absence of the *armA* gene in the pEX18 plasmid, which indicated that the *armA* gene may insert through the conservative transposable element carried by both p1632-2 and pCTX-M-3. p1632-2 plasmid was predicted to carry essential conjugative modules (*oriT*, relaxase, type IV coupling protein [T4CP], and T4SS). The results of the conjugation also confirmed that the p1632-2 plasmids could transfer to Escherichia coli J53 with high-conjugation frequencies (0.98 × 10^−1^ to 1.96 × 10^−1^) (Table 3).

**TABLE 3:** Conjugation frequency of resistant plasmids identified in K. pneumoniae 1632 and 1864

Plasmid	Resistance gene	No. of independent determinations	Conjugation frequencies	
			Mean	Range
p1632-2	*armA*	3	1.6 × 10^−1^	0.98 × 10^−1^ to 1.96 × 10^−1^
p1864-1	*armA*	3	9.5 × 10^−7^	8.56 × 10^−7^ to 10.44 × 10^−7^

### Resistance gene *armA* and virulence factor *iuc* operon could be cotransferred by conjugative p1864-1 plasmid.

**FIG 2:**
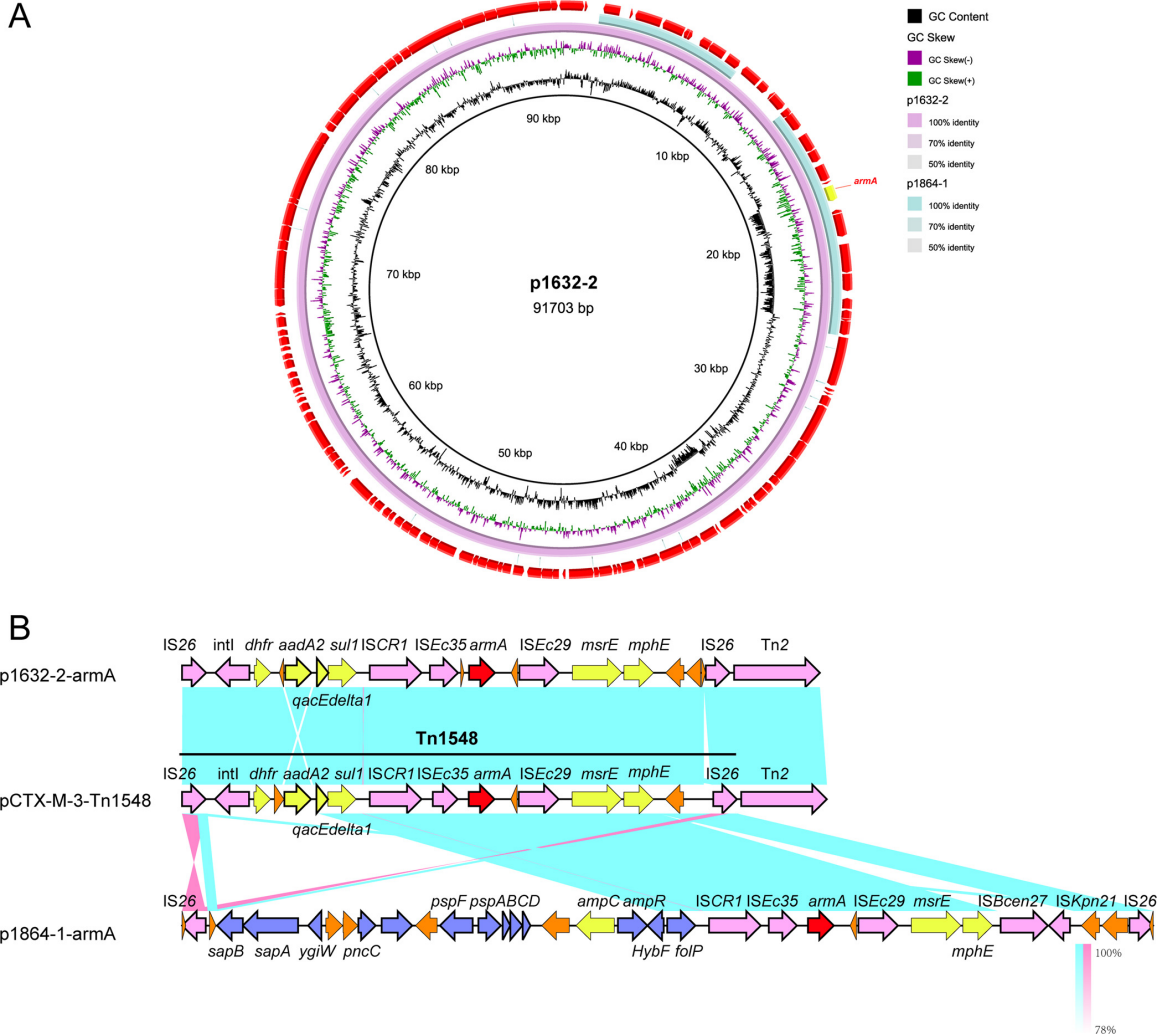
Comparative analysis of *rmtB*/*bla*_KPC-2_ positive p1632-1 and p1864-2 plasmids. (A) Comparison with three IncFII plasmids pl632-1, p1864-2, and reference plasmid pKPHS2. (B) Linear comparison of the *rmtB* and *bla*_KPC-2_ regions of p1632-1 and p1864-2 plasmids. The 16S-RMTase-encoding genes are shown in red, mobile genetic elements are shown in purple, other antimicrobial resistance genes are shown in yellow, and unidentified ORFs are shown in orange. Blue and pink shading indicates nucleotide identity.

**FIG 3:**
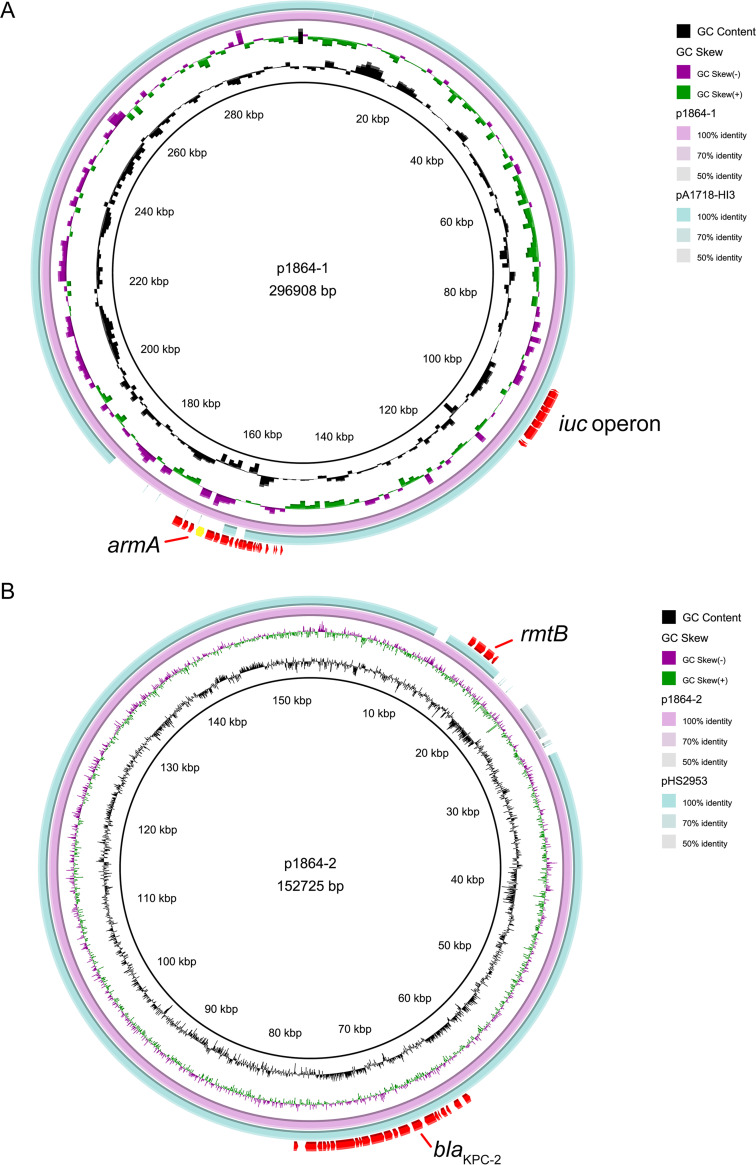
Comparative analysis of p1864-1 and p1864-2 plasmids with other reference plasmids. (A) Genome alignment was performed with p1864-1 (CP084493) and with another IncFIB virulent plasmid pA1718-HI3 (MW013142.1, *iuc* operon positive) (B) Genome alignment was performed with p1864-2 (CP084494) and with another IncFII plasmid pHS2953 (MT875328.1).

**FIG 4:**
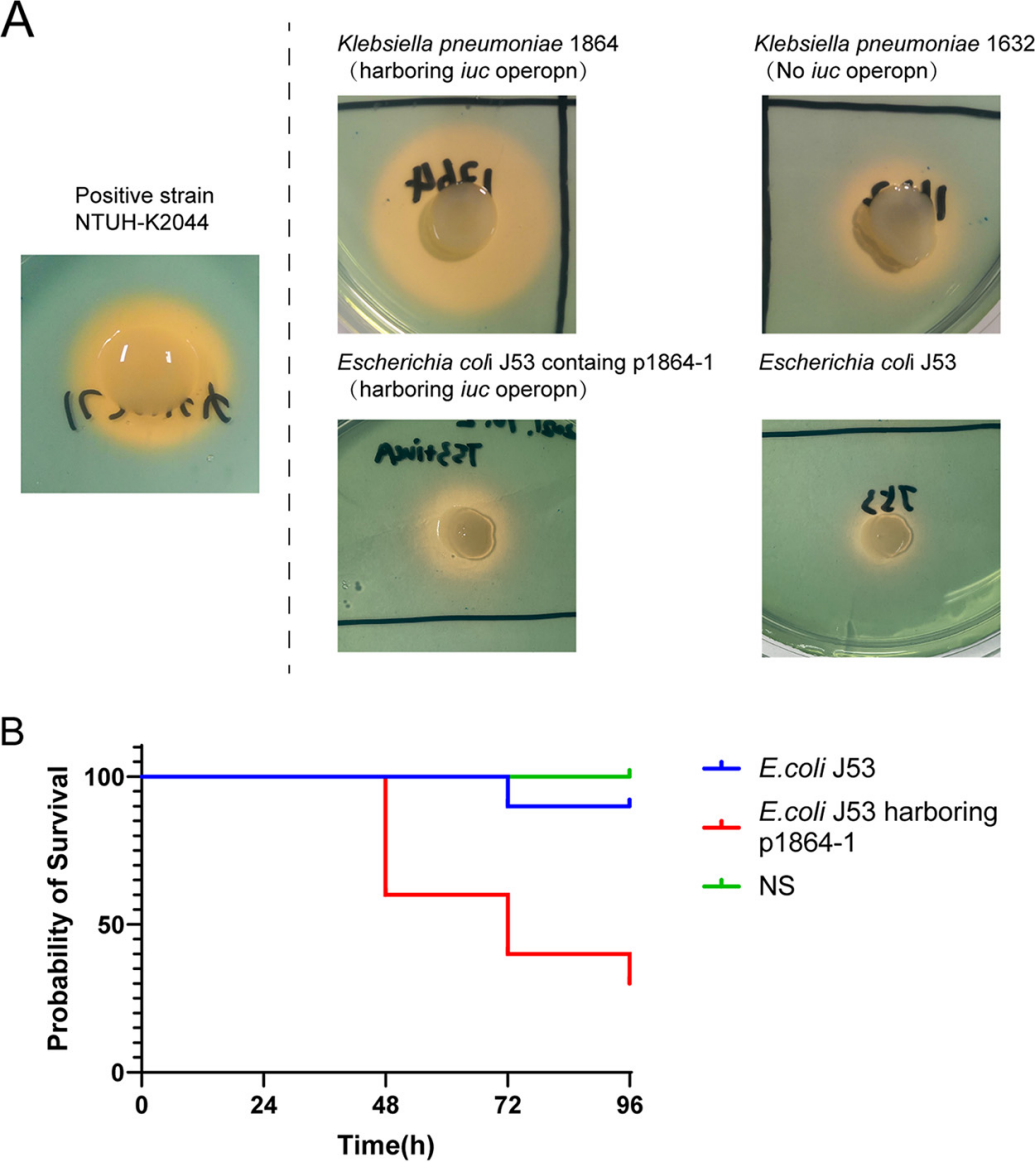
The virulence analysis of p1864-1 plasmid. (A) Siderophore production of K. pneumoniae and E. coli harboring or not harboring iuc-positive plasmid (p1864-1). Hypervirulent K. pneumoniae NTUH-K2044 was used as positive control. (B) Survival rates of G. mellonella infected with E. coli J53 harboring or not harboring iuc-positive plasmid (p1864-1). A log-rank (Mantel-638 Cox) test was performed for the indicated curves. A significant difference (*P < *0.0001) was observed between these groups. NS, normal saline.

We also observed *armA* plasmid, *rmtB*-*bla*_KPC-2_ plasmid, respectively, in K. pneumoniae 1864, but the genetic features were different from the two plasmids identified in K. pneumoniae 1632. The *armA*-positive plasmid p1864-1 (296,908 bp) was typed as IncFIB plasmid, harboring four completed conjugation modules and sharing a low identity with the IncM2 plasmid p1632-2 described above (Fig. 2). p1864-1 plasmid was highly identical to another IncFIB virulent plasmid pA1718-HI3 (MW013142.1, *iuc* operon positive); the main difference between these two IncFIB plasmids (p1864-1 and pA1718-HI3) was also the absence of *armA* in the pA1718-HI3 reference plasmid (Fig. 3). We found that p1864-1 (*armA* and *iuc*) could be effectively transferred to E. coli J53 (8.56 × 10^−7^-10.44 × 10^−7^) (Table 3). Notably, not only could the resistance phenotype be delivered, but also the virulence was cotransferred to the recipients. Siderophore production increased when the E. coli J53 obtained the p1864-1 plasmid, with the diameter of the halo increased ∼2-fold, and the acquirement of p1864-1 plasmid also significantly decreased the survival rate of Galleria mellonella (Fig. 4). All these findings highlight the threat of the transmissible p1864-1 plasmid.

**FIG 5:**
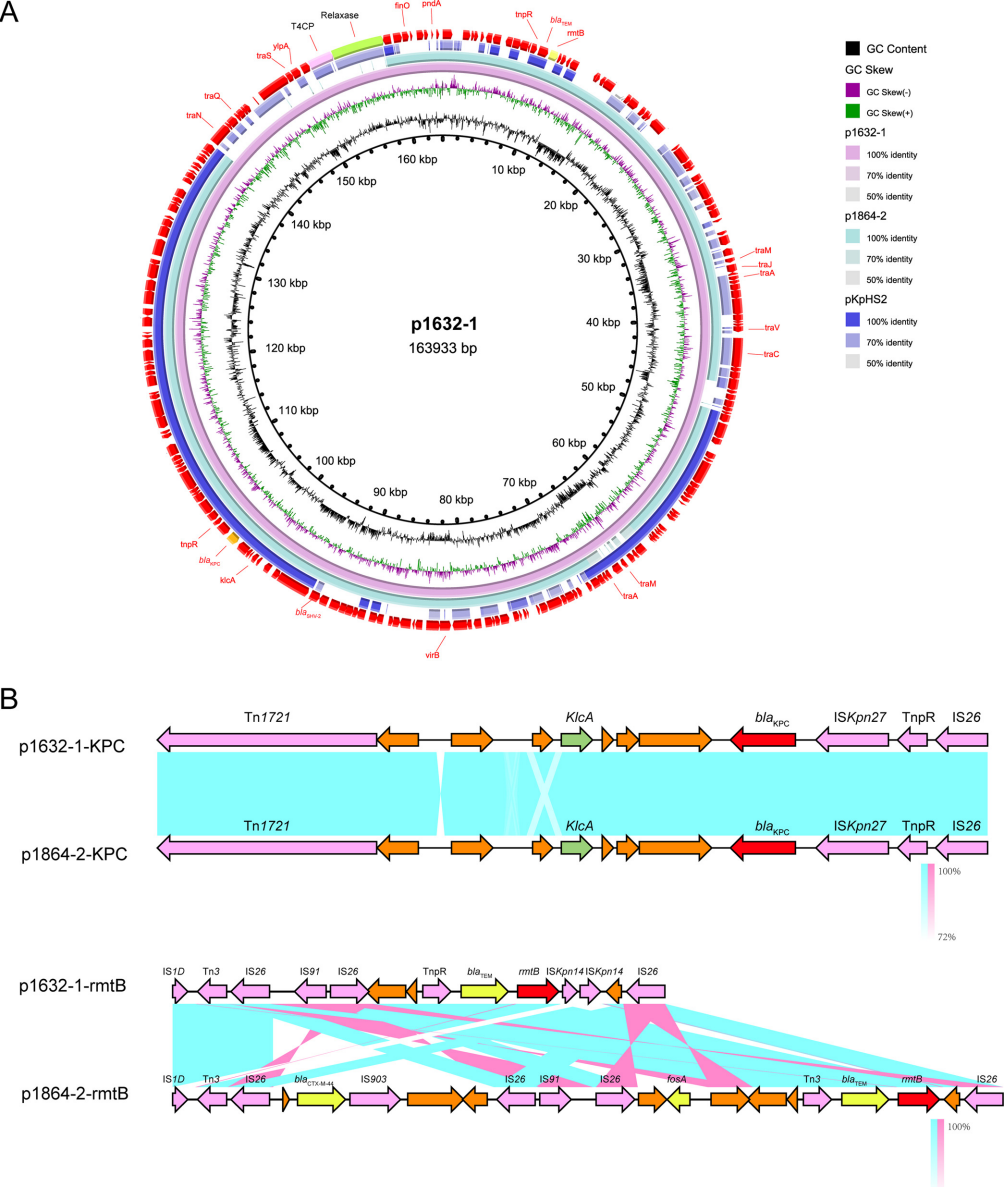
Comparative analysis of armA-positive p1632-2 and p1864-1 plasmids. (A) Comparison with plasmids pl632-2 and p1864-1. (B) Linear comparison of the armA regions of p1632-1 and p1864-2 plasmids. The 16S-RMTase-encoding genes are shown in red, mobile genetic elements are shown in purple, other antimicrobial resistance genes are shown in yellow, other annotated ORFs are shown in blue, and unidentified open reading frames (ORFs) are shown in orange. Blue and pink shading indicates nucleotide identity.

The *rmtB/bla*_KPC-2_-positive plasmid p1864-2 (152,725 bp) was also typed as IncFII plasmid; however, we did not observe this plasmid carrying the completed conjugation module, which lacked two key conjugation parts: relaxase and T4CP protein. These findings showed that p1864-2 could not self-transfer (Fig. 5). However, the *oriT* site found in p1864-2 plasmid provided the possibility for the mobilization of p1864-2 plasmid; thus, the dissemination threat of p1864-2 could not be ignored.

### Mobile genetic elements associated with *armA*, *rmtB*, and *bla*_KPC-2_ in K. pneumoniae 1632 and 1864 were polymorphic.

In addition to the plasmids, the actions of other MGEs, such as insertion sequences (ISs) and transposons (Tns) also contribute to the capture, accumulation, and dissemination of resistance genes. Hence, we also analyze the MGEs surrounding these three key resistant elements to evaluate the dissemination potential of these resistance genes in both K. pneumoniae 1632 and 1864.

Although the genetic identity of p1632-2 (*armA*-positive plasmid in K. pneumoniae 1632) and p1864-1 (*armA*-positive plasmid in K. pneumoniae 1864) was low, the genetic regions surrounding the *armA* gene were conserved in two plasmids, which were carried by complete Tn*1548* transposon closely relating to pCTX-M-3 (11). The complete Tn*1548* sequence was flanked by two IS*26* elements, together with several other mobile elements, such as *intI*, IS*CR1*, IS*Ec35* (IS*903* group), and ISEc29 (IS*10* group). The Tn*1548* transposon in p1864-1 plasmid was complex, in addition to the conservative IS*CR1*-IS*Ec35*-*armA*-ISEc29-*msrE-mphE* cluster, there are ∼20 open reading frames (ORFs) inserted around this cluster, with some elements associated with metabolic activity and other resistance elements (Fig. 2B). These results indicated that the movement of IS*26* could stimulate the evolution of *armA* plasmids.

Both p1632-1 and p1864-2 (*rmtB* and *bla*_KPC-2_ positive) plasmids were typed as IncFII/IncR plasmids, which is a major plasmid incompatibility group recognized in *Enterobacteriaceae* (15, 16). We compared these two plasmids with the classical IncFII/IncR plasmid pKPHS2 to analyze the divergency and conservative region in three plasmids (Fig. 5). We found that although the variance of the plasmid backbone existed, the *bla*_KPC-2_ cluster exhibited high identity between p1632-1 and p1864-2, belonging to the epidemic Tn*1721-bla*_KPC-2_ transposon (17). For another *rmtB*-associated antibiotic resistance island (ARI), the MGEs were polymorphic. In p1632-1, *rmtB* was linked with *bla*_TEM_, and *rmtB-bla*_TEM_ was flanked with eight mobile elements, including IS*1D*, IS*26*, IS*91*, IS*kpn14*, and Tn*3.* In p1864-2, the MGEs were also diverse and harbored more resistance determinants, which formed a *bla*_CTX-M-44_-*fosA3-bla*_TEM_-*rmtB* ARI (Fig. 5B). This ARI, together with multiple and diversity MGEs (IS*1D*, IS*26*, IS*91*, IS*903*, and Tn*3*), can form a highly active transmission among the strains, which is extremely harmful.

## DISCUSSION

Carbapenem-resistant K. pneumoniae (CRKP) have emerged as a global problem hindering the treatment of bacterial infections, since effective antibiotic treatments are limited (18). Previous studies showed that aminoglycosides are an available antibiotic option for the infections caused by CRKP and can lower the mortality rate effectively (4). Unfortunately, we observed two MDR K. pneumoniae that exhibited high-level resistance to both carbapenems and aminoglycosides, coharboring *armA*, *rmtB*, and *bla*_KPC_ resistance elements. Notably, we also identified a conjugative MDR virulent plasmid in such K. pneumoniae, which not only made the therapy tougher but also increased the transmission risk of such high-risk elements.

Both K. pneumoniae 1632 and 1864 isolated from the blood samples of sick patients hold similar resistant phenotypes and harbor similar resistant determinants. Previous studies indicated that the 16S-RMTases are strongly associated with carbapenemase-producing members of the Enterobacterales order (9), consistent with findings in K. pneumoniae 1632 and 1864.

In K. pneumoniae 1632, we got two resistant plasmids: p1632-1 (*bla*_KPC-2_ and *rmtB*) and p1632-2 (*armA*). Although p1632-1 harbored four conjugation modules, it lost the effective self-transferred ability. To form a successful conjugative process, the membrane-associated mating pair formation (MPF) complex, a form of T4SS providing the mating channel, is necessary (19). However, the p1632-1 plasmid lacked the essential protein (TraH and TraY) of T4SS_F_, leading to the failure of self-transmission. p1632-2 was another resistance plasmid harboring the *armA* gene. *armA* is a common element that conferred the high-level aminoglycoside phenotype that can be observed in the chromosome or multiple plasmids, belonging to different incompatibility groups (IncM2, IncFII, IncR, IncX1, and IncN) (20–24). In this study, the p1632-2 plasmid was typed as IncM2 plasmid and had an extremely high-conjugative frequency, higher than the other IncM2-*armA* plasmid pMS3802OXARMA studied before (10^−1^ *versus* 10^−4^) (25). As previous studies described (19, 26), the *oriT*-positive plasmid could be cotransferred with the conjugative plasmid, and the p1632-1 plasmid (*oriT* and relaxase protein positive) still holds the potential to be mobilized when p1632-2 is induced.

K. pneumoniae 1864 also held two resistance plasmids carrying *armA*, *rmtB*, and *bla*_KPC-2_, respectively. Both *rmtB* and *bla*_KPC-2_ also located on one IncFII plasmid (p1864-2), but the p1864-2 plasmid still held several discrepancies with p1632-1, especially on the conjugation region. Unlike the p1632-1 plasmid, the IncFII-p1864-2 plasmid did not contain four conjugative modules, which contained only the core *oriT* site and T4SS. Similar to the p1632-1 plasmid, although the self-transmission ability was absent in p1864-2 plasmid, it still typed as a mobilizable plasmid (*oriT* positive) (19). We also found another *armA*-positive plasmid, p1864-1, which is different from the p1632-2 (IncM2) plasmid identified in K. pneumoniae 1632. The p1864-1 plasmid was typed as IncFIB/IncH1B, the same type of epidemic virulent plasmid in K. pneumoniae (27). Notably, the p18464-1 plasmid was a typical MDR virulent plasmid, coharboring resistance elements (*armA*) and a virulence factor (*iuc* operon). Unlike conventional virulence plasmids retaining two important siderophore clusters (*iro* and *iuc*), p1864-1 harbored only *iucABCDiutA* genes. Although the *iro* were deleted, the reserved *iuc* clusters on plasmids were sufficient for conferring a hypervirulent phenotype (28). Moreover, siderophore production results and the survival rate of G. mellonella indicated that obtaining p1864-1 plasmid would promote the pathogenicity of K. pneumoniae. What is most frightening is that the p1864-1 not only exhibited the MDR-virulent phenotype but also can effectively self-transfer among clinical organisms. Such virulence factor co-occurred with multiple resistance elements in K. pneumoniae 1864, resulting in the hypervirulent (hv) MDR phenotype, which made treatment tougher.

Although the presence of *armA* or *rmtB* alone can mediate high levels of aminoglycoside resistance, the unusual coexistence of both elements in one K. pneumoniae still holds more threat than if only one determinant existed (29). Although the coexistence of *armA* and *rmtB* has been reported before (29), the *armA* gene was located on a chromosome, not on transmissible plasmids as observed in our study. The coexistence of transmissible resistance plasmids in one isolate could act as a vast storage pool of resistant elements. In both K. pneumoniae isolates analyzed in our study, *armA* was located on the effective self-transmissible plasmids, and these plasmids will transfer the high-level aminoglycoside resistance phenotype. *rmtB* and *bla*_KPC-2_ co-occurred on the mobilization plasmid, increasing the transmission of MDR features.

According to the analysis of the four resistant plasmids in K. pneumoniae 1632 and 1864, the plasmids extracted from these two isolates were different. For the *armA* plasmids, although the plasmid backbones of p1632-2 (IncM2) and p1864-1 (IncFIB) were different, the *armA* clusters shared high similarity, which was carried by the Tn*1548* transposase. This genetic environment has been reported to promote efficient *armA* mobilization in *Enterobacterales* (30). Moreover, IncM2 and IncFIB plasmids are thought to be broad-host-range plasmids, which could therefore mediate widespread dissemination. The genetic background of the *armA* gene is consistent with the spread of the Tn*1548* transposon and the plasmids in strains with different genetic backgrounds. For the *bla*_KPC-2_ cluster in p1632-1 and p1864-2, they were all located on the epidemic and classical Tn*1721* transposon, as described before (17). The most polymorphic MGEs were observed surrounding the *rmtB* elements; we observed eight mobile elements and diversity linking-resistant elements, which formed a large ARI and could be transferred and inserted into other isolates. Those diverse mobile structures in two isolates made a great contribution to the dissemination of resistance elements.

In this study, we report two MDR K. pneumoniae, which exhibited high-level resistance to both carbapenems and aminoglycosides simultaneously; we focused on the uncommon co-occurrence of *armA* and *rmtB*. The coexistence of these two aminoglycoside-related resistance genes would severely limit the therapy choices. Moreover, the co-occurrence of *rmtB* and *bla*_KPC-2_ on one mobilized plasmid and the location of *armA* on highly self-transferred conjugative plasmids resulted in a high spread risk for these resistance elements. Notably, the cotransferrence of *armA* and *iuc* in K. pneumoniae 1864 could promote rapid emergence of hv-MDR K. pneumoniae strains and augmented the virulence level of the host strain. Moreover, various mobile elements surrounding resistant genes greatly increased the risk of spread of these resistant phenotypes. Future studies are necessary to evaluate the prevalence of such multidrug-resistant isolates.

## MATERIALS AND METHODS

### Bacterial strains.

From July 2015 to December 2018, we collected 137 unique (one isolate per patient) CRKP isolates from patients with bloodstream infections (BSIs) in 11 tertiary China teaching hospitals to explore the prevalence of 16S rRNA methyltransferase genes (31). K. pneumoniae strain 1632 and 1864 were selected from them, for they are the only two isolates harboring the novel coexistence of *armA*, *rmtB*, and *bla*_KPC_. Plasmid conjugation was performed with E. coli J53 (sodium azide^R^), which was used as the recipient for the selection of related transconjugants.

### Antimicrobial susceptibility test.

The MIC of the original isolate 1632 and 1864 and all transconjugants were determined by broth microdilution following the Clinical and Laboratory Standards Institute guidelines. E. coli ATCC 25922 was used as a quality control strain for MIC determination. The interpretative breakpoints were based on CLSI2021-M100-ED31.

### Whole-genome sequencing and bioinformatics analysis.

The genomic DNA of 1632 and 1864 was extracted using a commercial DNA extraction kit (Qiagen, Germany) and the genome sequencing was then performed by the PacBio Sequel platform and the Illumina NovaSeq 6000 platform (the total number of bases of 1632 was 1,463,089,800 bp, and that of 1864 was 1,465,722,900 bp). Data assembly proceeded after adapter contamination removal and data filtering using AdapterRemoval (32) and SOAPec (33). The filtered reads were assembled by SPAdes (version 3.12) (34) and A5-miseq (version 20160825) (35) to construct scaffolds and contigs. CANU (version 1.7.1) (36) software was applied to assemble the data acquired by PacBio platform sequencing. Afterwards, whole assembled results were integrated to generate a complete sequence, and the genome sequence was obtained after correction using Pilon software. Resistant plasmid replicons were identified using the PlasmidFinder database using the minimum coverage and minimum identities of 90% (https://cge.cbs.dtu.dk/services/PlasmidFinder/). The acquired antibiotic resistance genes were identified using ResFinder (https://cge.cbs.dtu.dk/services/ResFinder/) with the default threshold. To determine whether the plasmids could self-transmission primarily, we used oriTfinder (https://tool-mml.sjtu.edu.cn/oriTfinder/oriTfinder.html) to conduct a detailed analysis of the conjugation module, including the origin of transfer site (*oriT*), the relaxase gene, the type IV coupling protein (T4CP) gene, and the type IV secretion system gene cluster (T4SS). The related insertion sequences (ISs) and transposons (Tns) were determined through the ISFinder (https://www-is.biotoul.fr/). The multilocus sequence typing (MLST) and the serotype were determined by the BIGSdb-Pasteur (https://bigsdb.pasteur.fr/cgi-bin/bigsdb/bigsdb.pl?db=pubmlst_klebsiella_seqdef) and Kleborate (version 0.3.0) (https://github.com/katholt/Kleborate/). BLAST Ring Image Generator (BRIG) was used to compare key resistant plasmids with other representative plasmids to further generate circular plasmid maps. Easyfig software was used to generate the comparison of the gene environments surrounding the vital resistant genes.

### Conjugation assay.

We applied conjugation assay to evaluate whether these resistant plasmids could be transferred from K. pneumoniae 1632 and 1864 (donor isolate) to E. coli J53 (recipient isolate). The donors and recipients were cultured in Luria-Bertani broth (37°C) to the logarithmic phase, mixed in a 1:1 ratio, centrifuged at 8,000 × *g* for 1 min, and then resuspended in 20 μL MgSO_4_ (10 mM). The resuspension was spotted on the Luria-Bertani (LB) plate and incubated at 37°C overnight. Subsequently, the serial dilutions were plated in media with appropriate antibiotics (meropenem [MEM], 1 mg/L [*bla*_KPC-2_]; gentamicin, 16 mg/L [*armA* and *rmtB*]; sodium azide, 100 mg/L [J53 recipient]). The conjugation frequency was calculated as the number of transconjugants per donor. All transconjugants were confirmed by PCR for the presence of the *armA*, *rmtB*, and *bla*_KPC-2_ genes. All the primers are listed in Table 4.

**TABLE 4:** Oligonucleotides for PCR

Name	Forward/reverse	Sequence
ArmA	Forward	TGGGGGTCTTACTATTCTGCC
	Reverse	CCATTCCCTTCTCCTTTCCAGA
RmtB	Forward	CCCCCAAACAGACCGTAGAG
	Reverse	GCCTAAACTACGCGTGGGAA
KPC-2	Forward	TCGCTAAACTCGAACAGG
	Reverse	TTACTGCCCGTTGACGCCCAATCC

### Quantitative siderophore production assay.

The chrome azurol S (CAS) assay was performed as previously described to determine the iron-chelating capabilities of bacterial supernatants (14). Briefly, the stationary-phase iron-chelated cultures (10 μL) were dropped on CAS plates, and siderophore production was determined by the orange halos after incubation for 48 h at 37°C.

### G. mellonella killing studies.

To evaluate the pathogenicity of the *iuc* plasmid extracted from K. pneumoniae strain1864, we applied the G. mellonella killing assays. G. mellonella caterpillars were stored at 4°C before use. Caterpillars were selected weighing 150 to 200 mg each. For each group, the caterpillars were inoculated with 10 μL of at a concentration of 3.75 × 10^8^ CFU/mL E.coli J53 or E. coli J53 harboring the *iuc* plasmid (p1864-1). The normal saline-treated group was set to be the control group. A minimum of 30 caterpillars were in each treatment group; they were kept in three petri dishes at 37°C and inspected daily for 4 days. The survival rates were recorded for each day.

### Nucleotide sequence accession numbers of K. pneumoniae 1632 and 1864.

The complete nucleotide sequences of the chromosome of K. pneumoniae 1632 and plasmids p1632-1, p1632-2, p1632-3, and p1632-4 were submitted to GenBank under accession numbers CP084497, CP084498, CP084499, CP084500, and CP084501, respectively. The complete nucleotide sequences of the chromosome of K. pneumoniae 1864 and plasmids p1864-1, p1864-2, p1864-3, and p1864-4 were submitted to GenBank under accession numbers CP084492, CP084493, CP084494, CP084495, and CP084496, respectively.

## References

[1] Tzouvelekis LS, Markogiannakis A, Psichogiou M, Tassios PT, Daikos GL. 2012. Carbapenemases in *Klebsiella pneumoniae* and other *Enterobacteriaceae*: an evolving crisis of global dimensions. Clin Microbiol Rev 25:682–707. doi:10.1128/CMR.05035-11. 23034326 PMC3485753

[2] Chen L, Mathema B, Chavda KD, DeLeo FR, Bonomo RA, Kreiswirth BN. 2014. Carbapenemase-producing *Klebsiella pneumoniae*: molecular and genetic decoding. Trends Microbiol 22:686–696. doi:10.1016/j.tim.2014.09.003. 25304194 PMC4365952

[3] Becker B, Cooper MA. 2013. Aminoglycoside antibiotics in the 21st century. ACS Chem Biol 8:105–115. doi:10.1021/cb3005116. 23110460

[4] Daikos GL, Tsaousi S, Tzouvelekis LS, Anyfantis I, Psichogiou M, Argyropoulou A, Stefanou I, Sypsa V, Miriagou V, Nepka M, Georgiadou S, Markogiannakis A, Goukos D, Skoutelis A. 2014. Carbapenemase-producing *Klebsiella pneumoniae* bloodstream infections: lowering mortality by antibiotic combination schemes and the role of carbapenems. Antimicrob Agents Chemother 58:2322–2328. doi:10.1128/AAC.02166-13. 24514083 PMC4023796

[5] Nasiri G, Peymani A, Farivar TN, Hosseini P. 2018. Molecular epidemiology of aminoglycoside resistance in clinical isolates of *Klebsiella pneumoniae* collected from Qazvin and Tehran provinces, Iran. Infect Genet Evol 64:219–224. doi:10.1016/j.meegid.2018.06.030. 29964191

[6] Miró E, Grünbaum F, Gómez L, Rivera A, Mirelis B, Coll P, Navarro F. 2013. Characterization of aminoglycoside-modifying enzymes in *Enterobacteriaceae* clinical strains and characterization of the plasmids implicated in their diffusion. Microb Drug Resist 19:94–99. doi:10.1089/mdr.2012.0125. 23206280

[7] Poole K. 2004. Efflux-mediated multiresistance in Gram-negative bacteria. Clin Microbiol Infect 10:12–26. doi:10.1111/j.1469-0691.2004.00763.x. 14706082

[8] Doi Y, Wachino J-I, Arakawa Y. 2016. Aminoglycoside resistance: the emergence of acquired 16S ribosomal RNA methyltransferases. Infect Dis Clin North Am 30:523–537. doi:10.1016/j.idc.2016.02.011. 27208771 PMC4878400

[9] Kawai A, Suzuki M, Tsukamoto K, Minato Y, Doi Y. 2021. Functional and structural characterization of acquired 16S rRNA methyltransferase NpmB1 conferring pan-aminoglycoside resistance. Antimicrob Agents Chemother 65:e0100921. doi:10.1128/AAC.01009-21. 34310216 PMC8448102

[10] Yu FY, Yao D, Pan JY, Chen C, Qin ZQ, Parsons C, Yang LH, Li QQ, Zhang XQ, Qu D, Wang LX. 2010. High prevalence of plasmid-mediated 16S rRNA methylase gene rmtB among *Escherichia coli* clinical isolates from a Chinese teaching hospital. BMC Infect Dis 10:184. doi:10.1186/1471-2334-10-184. 20573216 PMC2905422

[11] Caméléna F, Morel F, Merimèche M, Decousser JW, Jacquier H, Clermont O, Darty M, Mainardis M, Cambau E, Tenaillon O, Denamur E, Berçot B, IAME Resistance Group. 2020. Genomic characterization of 16S rRNA methyltransferase-producing *Escherichia coli* isolates from the Parisian area, France. J Antimicrob Chemother 75:1726–1735. doi:10.1093/jac/dkaa105. 32300786

[12] Nagasawa M, Kaku M, Kamachi K, Shibayama K, Arakawa Y, Yamaguchi K, Ishii Y. 2014. Loop-mediated isothermal amplification assay for 16S rRNA methylase genes in Gram-negative bacteria. J Infect Chemother 20:635–638. doi:10.1016/j.jiac.2014.08.013. 25179393

[13] Zhang X, Li Q, Lin H, Zhou W, Qian C, Sun Z, Lin L, Liu H, Lu J, Lin X, Li K, Xu T, Zhang H, Li C, Bao Q. 2021. High-level aminoglycoside resistance in human clinical *Klebsiella pneumoniae* complex isolates and characteristics of armA-carrying IncHI5 plasmids. Front Microbiol 12:636396. doi:10.3389/fmicb.2021.636396. 33897641 PMC8058188

[14] Tian D, Wang W, Li M, Chen W, Zhou Y, Huang Y, Sheng Z, Jiang X. 2021. Acquisition of the conjugative virulence plasmid from a CG23 hypervirulent *Klebsiella pneumoniae* strain enhances bacterial virulence. Front Cell Infect Microbiol 11:752011. doi:10.3389/fcimb.2021.752011. 34604119 PMC8485780

[15] Bi D, Zheng J, Li J, Sheng Z, Zhu X, Ou H, Li Q, Wei Q. 2018. *In silico* typing and comparative genomic analysis of IncFIIK plasmids and insights into the evolution of replicons, plasmid backbones, and resistance determinant profiles. Antimicrob Agents Chemother 62:e00764-18. doi:10.1128/AAC.00764-18. PMC6153814 30012771

[16] Fu P, Tang Y, Li G, Yu L, Wang Y, Jiang X. 2019. Pandemic spread of blaKPC-2 among *Klebsiella pneumoniae* ST11 in China is associated with horizontal transfer mediated by IncFII-like plasmids. Int J Antimicrob Agents 54:117–124. doi:10.1016/j.ijantimicag.2019.03.014. 30885806

[17] Tang Y, Li G, Liang W, Shen P, Zhang Y, Jiang X. 2017. Translocation of carbapenemase gene *bla*_KPC-2_ both internal and external to transposons occurs via novel structures of Tn1721 and exhibits distinct movement patterns. Antimicrob Agents Chemother 61:e01151-17. doi:10.1128/AAC.01151-17. 28784666 PMC5610484

[18] Doi Y. 2019. Treatment options for carbapenem-resistant Gram-negative bacterial infections. Clin Infect Dis 69:S565–S575. doi:10.1093/cid/ciz830. 31724043 PMC6853760

[19] Smillie C, Garcillán-Barcia MP, Francia MV, Rocha EPC, de la Cruz F. 2010. Mobility of plasmids. Microbiol Mol Biol Rev 74:434–452. doi:10.1128/MMBR.00020-10. 20805406 PMC2937521

[20] Taylor E, Bal AM, Balakrishnan I, Brown NM, Burns P, Clark M, Diggle M, Donaldson H, Eltringham I, Folb J, Gadsby N, Macleod M, Ratnaraja N, Williams C, Wootton M, Sriskandan S, Woodford N, Hopkins KL. 2021. A prospective surveillance study to determine the prevalence of 16S rRNA methyltransferase-producing Gram-negative bacteria in the UK. J Antimicrob Chemother 76:2428–2436. doi:10.1093/jac/dkab186. 34142130

[21] Wangkheimayum J, Bhattacharjee M, Das BJ, Singha KM, Chanda DD, Bhattacharjee A. 2020. Expansion of acquired 16S rRNA methytransferases along with CTX-M-15, NDM and OXA-48 within three sequence types of *Escherichia coli* from northeast India. BMC Infect Dis 20:544. doi:10.1186/s12879-020-05264-4. 32711470 PMC7382822

[22] Oshiro S, Tada T, Watanabe S, Tohya M, Hishinuma T, Uchida H, Kuwahara-Arai K, Mya S, Zan KN, Kirikae T, Tin HH. 2020. Emergence and spread of carbapenem-resistant and aminoglycoside-panresistant *Enterobacter cloacae* complex isolates coproducing NDM-type metallo-β-lactamase and 16S rRNA methylase in Myanmar. mSphere 5:e00054-20. doi:10.1128/mSphere.00054-20. 32161144 PMC7067590

[23] Gajamer VR, Bhattacharjee A, Paul D, Ingti B, Sarkar A, Kapil J, Singh AK, Pradhan N, Tiwari HK. 2020. High prevalence of carbapenemase, AmpC β-lactamase and aminoglycoside resistance genes in extended-spectrum β-lactamase-positive uropathogens from Northern India. J Glob Antimicrob Resist 20:197–203. doi:10.1016/j.jgar.2019.07.029. 31398493

[24] Taylor E, Sriskandan S, Woodford N, Hopkins KL. 2018. High prevalence of 16S rRNA methyltransferases among carbapenemase-producing *Enterobacteriaceae* in the UK and Ireland. Int J Antimicrob Agents 52:278–282. doi:10.1016/j.ijantimicag.2018.03.016. 29596903

[25] Hernández M, López-Urrutia L, Abad D, De Frutos SM, Ocampo-Sosa AA, Eiros JM. 2021. First report of an extensively drug-resistant ST23 *Klebsiella pneumoniae* of capsular serotype K1 co-producing CTX-M-15, OXA-48 and ArmA in Spain. Antibiotics 10:157. doi:10.3390/antibiotics10020157. 33557209 PMC7913926

[26] Xu Y, Zhang J, Wang M, Liu M, Liu G, Qu H, Liu J, Deng Z, Sun J, Ou HY, Qu J. 2021. Mobilization of the nonconjugative virulence plasmid from hypervirulent *Klebsiella pneumoniae*. Genome Med 13:119. doi:10.1186/s13073-021-00936-5. 34294113 PMC8299605

[27] Tian D, Wang M, Zhou Y, Hu D, Ou HY, Jiang X. 2021. Genetic diversity and evolution of the virulence plasmids encoding aerobactin and salmochelin in *Klebsiella pneumoniae*. Virulence 12:1323–1333. doi:10.1080/21505594.2021.1924019. 33970792 PMC8115583

[28] Yang X, Dong N, Chan EW, Zhang R, Chen S. 2021. Carbapenem resistance-encoding and virulence-encoding conjugative plasmids in *Klebsiella pneumoniae*. Trends Microbiol 29:65–83. doi:10.1016/j.tim.2020.04.012. 32448764

[29] Yu F, Wang L, Pan J, Yao D, Chen C, Zhu T, Lou Q, Hu J, Wu Y, Zhang X, Chen Z, Qu D. 2009. Prevalence of 16S rRNA methylase genes in *Klebsiella pneumoniae* isolates from a Chinese teaching hospital: coexistence of rmtB and armA genes in the same isolate. Diagn Microbiol Infect Dis 64:57–63. doi:10.1016/j.diagmicrobio.2009.01.020. 19232867

[30] Galimand M, Sabtcheva S, Courvalin P, Lambert T. 2005. Worldwide disseminated *armA* aminoglycoside resistance methylase gene is borne by composite transposon Tn1548. Antimicrob Agents Chemother 49:2949–2953. doi:10.1128/AAC.49.7.2949-2953.2005. 15980373 PMC1168633

[31] Shen X, Liu L, Yu J, Ai W, Cao X, Zhan Q, Guo Y, Wang L, Yu F. 2020. High prevalence of 16S rRNA methyltransferase genes in carbapenem-resistant *Klebsiella pneumoniae* clinical isolates associated with bloodstream infections in 11 Chinese teaching hospitals. Infect Drug Resist 13:2189–2197. doi:10.2147/IDR.S254479. 32764995 PMC7367928

[32] Lindgreen S. 2012. AdapterRemoval: easy cleaning of next-generation sequencing reads. BMC Res Notes 5:337. doi:10.1186/1756-0500-5-337. 22748135 PMC3532080

[33] Luo R, Liu B, Xie Y, Li Z, Huang W, Yuan J, He G, Chen Y, Pan Q, Liu Y, Tang J, Wu G, Zhang H, Shi Y, Liu Y, Yu C, Wang B, Lu Y, Han C, Cheung DW, Yiu SM, Peng S, Xiaoqian Z, Liu G, Liao X, Li Y, Yang H, Wang J, Lam TW, Wang J. 2012. SOAPdenovo2: an empirically improved memory-efficient short-read *de novo* assembler. Gigascience 1:18. doi:10.1186/2047-217X-1-18. 23587118 PMC3626529

[34] Bankevich A, Nurk S, Antipov D, Gurevich AA, Dvorkin M, Kulikov AS, Lesin VM, Nikolenko SI, Pham S, Prjibelski AD, Pyshkin AV, Sirotkin AV, Vyahhi N, Tesler G, Alekseyev MA, Pevzner PA. 2012. SPAdes: a new genome assembly algorithm and its applications to single-cell sequencing. J Comput Biol 19:455–477. doi:10.1089/cmb.2012.0021. 22506599 PMC3342519

[35] Coil D, Jospin G, Darling AE. 2015. A5-miseq: an updated pipeline to assemble microbial genomes from Illumina MiSeq data. Bioinformatics 31:587–589. doi:10.1093/bioinformatics/btu661. 25338718

[36] Koren S, Walenz BP, Berlin K, Miller JR, Bergman NH, Phillippy AM. 2017. Canu: scalable and accurate long-read assembly via adaptive k-mer weighting and repeat separation. Genome Res 27:722–736. doi:10.1101/gr.215087.116. 28298431 PMC5411767

